# Complete Genome Sequence of Enterotoxigenic Escherichia coli Siphophage LL5

**DOI:** 10.1128/MRA.00674-19

**Published:** 2019-07-03

**Authors:** Denish Piya, Lauren Lessor, Mei Liu, Jason J. Gill

**Affiliations:** a Department of Biochemistry & Biophysics, Texas A&M University, College Station, Texas, USA; b Center for Phage Technology, Texas A&M University, College Station, Texas, USA; c Department of Animal Science, Texas A&M University, College Station, Texas, USA; DOE Joint Genome Institute

## Abstract

Here, we describe the complete genome sequence of siphophage LL5. LL5 is a T1-like phage isolated against enterotoxigenic Escherichia coli, which causes traveler’s diarrhea. LL5 is included as a component phage in the commercial prebiotic product PreforPro.

## ANNOUNCEMENT

Enterotoxigenic Escherichia coli (ETEC) strains are characterized by the presence of heat-labile enterotoxins (LT) and/or heat-stable enterotoxins (HT) (1). ETEC strains can induce traveler’s diarrhea (TD), resulting in watery diarrhea (1) which may be accompanied by nausea, vomiting, abdominal pain, fever, or blood in stool (2). TD typically self-resolves or may be successfully treated with antibiotics, but the global increase in the emergence of antibiotic resistance warrants the evaluation of alternative antibacterial approaches, such as the use of phages (3).

Phage LL5 was isolated by the enrichment method (4) from filter-sterilized (0.22-μm) influent from a municipal water treatment facility in College Station, TX, against a clinical ETEC isolate. Phage LL5 is also able to infect E. coli K-12 strains and was propagated on the E. coli strain DH5-alpha. The phage DNA was purified from high-titer lysates by a modified Wizard DNA purification kit (Promega) as previously described (5). Phage LL5 DNA was prepared using the GS FLX Titanium general DNA library preparation kit and was sequenced by FLX Titanium 454 pyrosequencing at the Emory-Georgia Research Alliance Genomics Center (Emory University, GA); trimmed sequence reads were assembled into a single contig at 19.9-fold coverage using Newbler version 2.5.3 (454 Life Sciences) at default settings. The contig was closed, based on its circular assembly, which produced identical sequences at each end. Structural annotation was conducted using GLIMMER version 3.0 (6) and MetaGeneAnnotator version 1.0 (7), with tRNAs predicted by ARAGORN version 2.36 (8) and verified by tRNAscan-SE (9) and gene functions predicted by InterProScan version 5.15-54.0 (10), the NCBI Conserved Domain Database (11), TMHMM version 2.0 (http://www.cbs.dtu.dk/services/TMHMM), BLASTp version 2.2.28 (12), and HHpred version 2.1 (13). Phage genome annotation was conducted using the Phage Galaxy (11) and Web Apollo (12) instances hosted by the Center for Phage Technology (https://cpt.tamu.edu). Phages were imaged by transmission electron microscopy at the Texas A&M University Microscopy and Imaging Center as previously described (14, 15).

LL5 is a siphophage with a head diameter of ∼60 nm and a flexible tail ∼150 nm long. LL5 has a genome of 49,788 bp with 42.5% G+C content, 88 predicted protein-coding genes, and no tRNA genes. Phage LL5 is closely related to the T1-like coliphage TLS (NCBI reference sequence NC_009540) (16), with 96% DNA sequence identity over 90% of the LL5 genome based on BLASTn. The genome produced a circular assembly and was reopened to be syntenic with other T1-like phages in the NCBI database, such as TLS (NC_009540) and T1 (NC_005833). Thirty-three LL5-encoded proteins could be assigned putative functions. Genes identified included a DNA primase/helicase (gp58), ATP-dependent helicase (gp60), and helicase (gp75). Structural proteins identified included the portal protein (gp31), major capsid protein (gp36), minor tail proteins (gp41, gp42, gp47, and gp48), tail tube protein (gp43), tape measure protein (gp46), and tail fiber proteins (gp51 and gp57). The small and large terminase subunits were identified as gp29 and gp30, respectively. Like its T1-like relatives, LL5 encodes a canonical lysis cassette composed of a holin (gp70), endolysin (gp71), and unimolecular spanin (gp72). Phage LL5 is currently a component of the prebiotic product PreforPro.

### Data availability.

The annotated phage genome is deposited in NCBI GenBank under accession no. MH491968. The associated BioProject, SRA, and BioSample accession numbers are PRJNA222858, SRR9134807, and SAMN11874326, respectively.
